# Completion Pancreaticoduodenectomy for Hereditary Pancreatitis After Prior Puestow Procedure: A Case Report

**DOI:** 10.1089/pancan.2018.0012

**Published:** 2018-09-25

**Authors:** James R. Nellen, Adam G. Strickland, Charles J. Yeo

**Affiliations:** Department of Surgery, Jefferson Pancreas, Biliary and Related Cancer Center, Thomas Jefferson University, Philadelphia, Pennsylvania.

**Keywords:** completion pancreatectomy, ethanol nerve block, hereditary pancreatitis, pancreaticoduodenectomy, Puestow

## Abstract

**Background:** Hereditary pancreatitis (HP) is an uncommon condition resulting from an imbalance of pancreatic proteases. Most commonly, protease serine 1 genetic mutations are causative for HP and often result in recurrent early onset episodes of acute pancreatitis typically progressing to chronic pancreatitis, with a high risk of pancreatic cancer.

**Case Presentation:** A 46-year-old female with HP, confirmed by genetic testing, presented with a 7-month history of recurrent pancreatitis. She had previously undergone a distal pancreatectomy and Puestow procedure in 1992 at 21 years of age, after having pancreatitis as a teenager. The patient now had a completion pancreaticoduodenectomy and celiac ethanol nerve block.

**Conclusion:** A completion pancreatectomy in patients with HP can be performed after previous pancreatic surgical intervention to treat disease manifestations and as a prophylaxis against an increased risk of pancreatic adenocarcinoma.

## Introduction

Hereditary pancreatitis (HP) is an uncommon condition leading to recurrent episodes of acute pancreatitis frequently resulting in a clinical picture of chronic pancreatitis. Often initially misdiagnosed as idiopathic pancreatitis, HP presents earlier than typical acute pancreatitis caused by alcohol or gallstones, and also carries an increased risk of pancreatic adenocarcinoma.^1–3^ Clinical presentation results from a pathological activation of cationic trypsinogen in the pancreatic parenchyma and genetic mutation in protease serine 1 (*PRSS1*), serine protease inhibitor Kazal type 1 (*SPINK1*), cystic fibrosis transmembrane conductance receptor (*CFTR*), and chymotrypsin C (*CTRC*) are commonly present.^3–5^ Many HP patients with acute pancreatitis will progress to chronic pancreatitis.^3,6^ Although a diagnosis of HP may be made clinically, advances in genetic testing have identified numerous *PRSS1* genetic mutations resulting in HP.^3,7^ In this report, we present a case of HP successfully managed with a completion pancreatectomy after previous pancreatic surgical intervention.

## Case Presentation

**FIG. 1.**
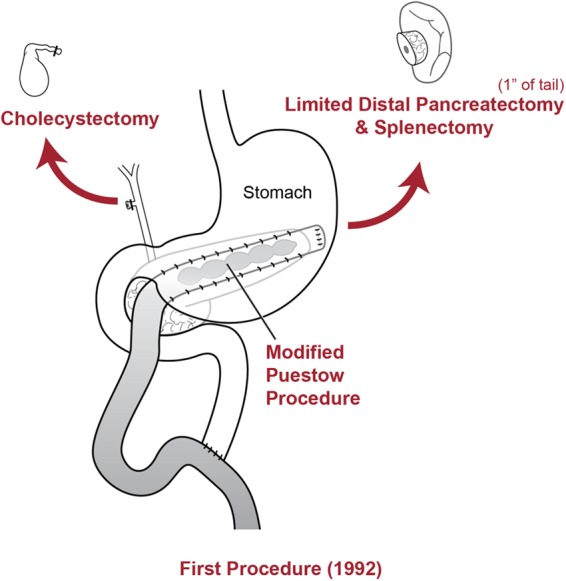
Diagram of the initial modified Puestow procedure.

**FIG. 2.**
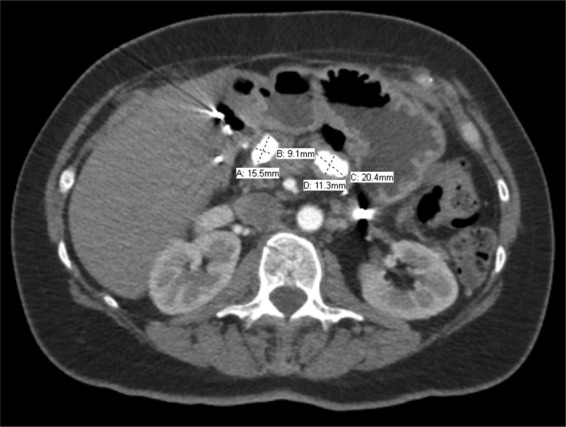
Computed tomography image showing two substantial calcifications in the pancreatic remnant and their measured size. Note the absence of the spleen in the left upper quadrant.

**FIG. 3.**
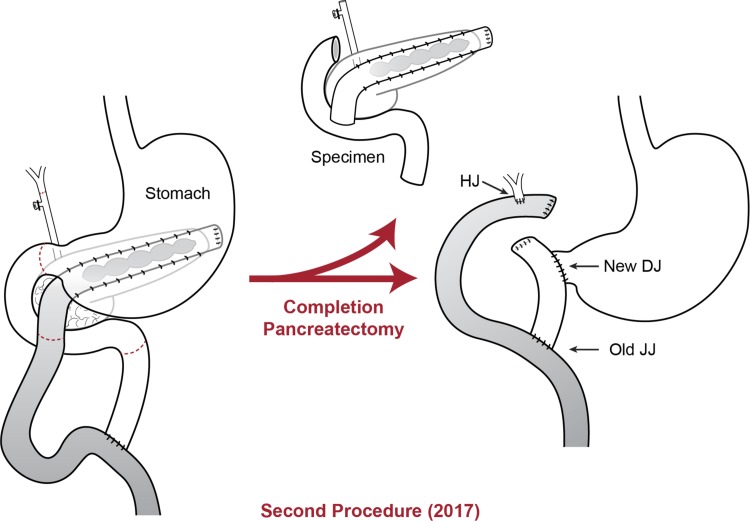
Diagram of the completion pancreaticoduodenectomy procedure. DJ, duodenojejunostomy; HJ, hepaticojejunostomy, JJ, jejunojejunostomy.

A 46-year-old Caucasian female presented in November of 2017 with a longstanding history of pancreatitis. The patient is part of a family with multiple diagnosed cases of pancreatitis who had undergone genetic testing to reveal a *PRSS1* gene mutation (N29I) and disease-modifying *CFTR* mutation ((TG)11-5T). The patient first showed symptoms of pancreatitis when she was 13 years old. Eight years later in 1992, the patient underwent a distal pancreatectomy, splenectomy, and cholecystectomy with modified Puestow procedure for symptoms of radiating epigastric pain, nausea, and vomiting typically lasting for 1 week and occurring three to four times per year (Fig. 1). Since that time, the patient had been followed every 6 months with accepted routine pancreatic cancer surveillance through magnetic resonance imaging and endoscopic ultrasonography.^8^ The patient did well until May of 2017 when she began experiencing recurring symptoms of acute pancreatitis, including dull waxing and waning epigastric pain with foods and liquids. On computed tomography scan, significant calcification in the right-sided pancreatic remnant was present (Fig. 2). Both CA 19-9 and carcinoembryonic antigen (CEA) values were elevated at 54 and 5.0, respectively. The patient elected to proceed with a completion pancreaticoduodenectomy and ethanol nerve block. Intraoperatively, there were significant adhesions present, which were lysed. The Puestow was taken down by transecting the Roux limb using a gastrointestinal anastomosis (GIA) stapler. The duodenum just distal to the pylorus was also transected using a GIA stapler in the same manner that is done during a pylorus-preserving pancreaticoduodenectomy. The hepaticojejunostomy was planned using the Roux limb from the prior Puestow procedure. The duodenojejunostomy was made with the proximal jejunum just distal to the ligament of Trietz (Fig. 3). The nerve block was performed by injecting 20 mL of 50% ethanol solution on either side of the aorta at the level of the celiac axis. The pathology on the resection specimen demonstrated chronic pancreatitis with parenchymal calcifications and duct ectasia consistent with *PRSS1*-related HP. No cancer was seen. The patient recovered well, was discharged on postoperative day 5, and was seen in follow-up fully recovered.

## Discussion

HP is an important cause of childhood pancreatitis, with a median age of symptom onset reported as ranging from 5 to 10 years and with a median age of diagnosis reported as ranging from 6 to 19 years.^1,2^ HP is most easily distinguished from non-HP in two ways: HP presents earlier in life, commonly in the first or early second decade of life, and it carries an increased risk of pancreatic adenocarcinoma (standardized incidence ratio 87).^1,3,9^ Despite an earlier age of onset, the hereditary nature of HP often goes undiagnosed. Nearly 50% and 75% of children diagnosed with acute recurrent pancreatitis and chronic pancreatitis, respectively, have an underlying genetic predisposition to their disease.^4^ The prevalence of HP has been estimated to be 0.3/100,000 people in France.^1^ Numerous gene mutations have been identified as causative or disease modifying for HP, the four most common being *PRSS1*, *SPINK1*, *CFTR*, and *CTRC*^3–5^ (Table 1). As already mentioned, the disease course of HP typically mirrors that of recurrent acute pancreatitis often culminating in a clinical picture of chronic pancreatitis. It should, however, be noted that some patients first present with chronic pancreatitis devoid of previous acute pancreatitis symptoms.^10^ Features of HP typically include classic pancreatic pain, nausea, and vomiting. In addition, pancreatic calcifications, parenchymal pseudocysts, and ductal abnormalities may be seen, often culminating in cholestasis, diabetes mellitus, and steatorrhea.^1^ Repeated episodes of pancreatitis often result in frequent interactions between medical providers and patients with HP.^10^ Treatment regimes include mitigation of risk factors (e.g., smoking and alcohol use), medical management through pancreatic enzyme supplementation and pain control, endoscopic pancreatic duct decompression, and surgical therapy. Treatment considerations must include the increased relative and absolute risk of pancreatic adenocarcinoma in patients with HP where the median age of diagnosis is 55 years.^1,9^

**Table 1.** **Common Gene Mutations in Hereditary Pancreatitis**

Gene	Cr	Inheritance	Disease relationship	Mutation
*PRSS1*	7*q34*	Autosomal dominant	Disease causing	Gain of function
*SPINK1*	5q32	Complex (most commonly autosomal recessive)	Usually disease modifying	Loss of function
*CFTR*	7q31.2	Autosomal recessive	Disease modifying	Loss of function
*CTRC*	1p36.21	Unreported	Disease modifying	Loss of function

## Conclusion

A completion pancreatectomy in patients with HP can be performed after previous pancreatic surgical intervention to treat disease manifestations and as a prophylaxis against the increased risk of pancreatic adenocarcinoma.

## Abbreviations Used

*CFTR*: cystic fibrosis transmembrane conductance receptor
*CTRC*: chymotrypsin C
*HP*: hereditary pancreatitis
*PRSS1*: protease serine 1
*SPINK1*: serine protease inhibitor Kazal type 1
